# Purtscher’s Retinopathy Following Long Bone Fracture: A Case Report

**DOI:** 10.7759/cureus.53087

**Published:** 2024-01-28

**Authors:** Muzzaffar Mad Isa, Yaakub Azhany, Rosnita Alias, Wan-Hazabbah Wan Hitam

**Affiliations:** 1 Department of Ophthalmology and Visual Science, School of Medical Sciences, Universiti Sains Malaysia, Kubang Kerian, MYS; 2 Department of Ophthalmology, Hospital Sultan Abdul Halim, Sungai Petani, MYS

**Keywords:** cotton wool spots, purtscher flecken, retina ischaemia, long bone fracture, purtscher’s retinopathy

## Abstract

Purtscher's retinopathy represents an occlusive retinal microvasculopathy that poses a potential threat to vision and is linked to traumatic events. This condition typically manifests in individuals following trauma, commonly associated with long bone fractures, head injuries, or thoracic compression. We report a rare case of unilateral Purtscher’s retinopathy after sustaining a long bone fracture. A 27-year-old healthy man sustained an open, comminuted midshaft fracture of the right femur after an alleged motor vehicle accident. On day 3 post trauma, he developed sudden right eye painless reduced vision. Visual acuity in the right eye was 6/12 pinhole 6/12 and the left eye was 6/9 pinhole 6/6. The pupillary reflex was normal in both eyes. Both anterior segments were unremarkable. Fundoscopy showed the presence of multiple cotton wool spots and fleckens in the right eye. Macula optical coherence tomography of the right eye confirmed hyperreflective lesions within the retinal nerve fiber layer. He was diagnosed with Purtscher’s retinopathy. The patient was treated conservatively given the fairly good visual acuity. There was complete resolution of fundus lesions with good visual acuity of 6/6 after one month. Ophthalmologic evaluation is crucial in cases of post-traumatic visual impairment, particularly in scenarios involving long bone fractures, to effectively exclude the possibility of Purtscher's retinopathy.

## Introduction

Purtscher's retinopathy represents a potentially vision-threatening occlusive retinal microvasculopathy linked to traumatic incidents. It is generally manifested in individuals post-trauma, primarily originating from occurrences such as thoracic compression or head injuries [1,2]. The designation "Purtscher-like retinopathies," conversely, pertains to individuals presenting with this condition in the absence of a trauma history, but in the presence of severe medical conditions such as renal failure, pancreatitis, and autoimmune diseases [2]. Initially documented by Purtscher in 1910, this ocular phenomenon was first observed in a man who experienced profound head trauma, subsequently encountering transient visual impairment. Purtscher identified distinctive retinal manifestations, specifically retinal hemorrhage and 'flecken' at the posterior pole of the patient's eyes. The pathogenesis of these pathological alterations was correlated by Purtscher with the extravasation of lymph from retinal vessels, attributed to elevated intracranial pressure after head trauma [3,4]. The prevalence of Purtscher and Purtscher-like retinopathies is projected to be approximately 0.24 per million annually; however, it is conceivable that these figures may be conservative, given the limited number of patients seeking ophthalmological evaluation for these manifestations [5,6]. Up to 60% of cases exhibit a bilateral onset [7]. It is rarely reported following long bone fractures.

We report a rare case of unilateral Purtscher’s retinopathy after sustaining a long bone fracture.

## Case presentation

A healthy 27-year-old male sustained an open comminuted fracture of the right femur following a motor vehicle accident. The patient underwent uneventful, emergency open reduction internal fixation surgery. On the third day post-trauma, he experienced the sudden onset of a painless generalized reduction in vision in the right eye. His description highlighted a central, foggy vision without progressive deterioration, devoid of aggravating or relieving factors. The patient denied experiencing central scotoma, photophobia, floaters, or flashes of light. Notably, there were no associated instances of head injury, blunt thoracic or abdominal trauma, or direct trauma to the ocular region.

**Figure 1:**
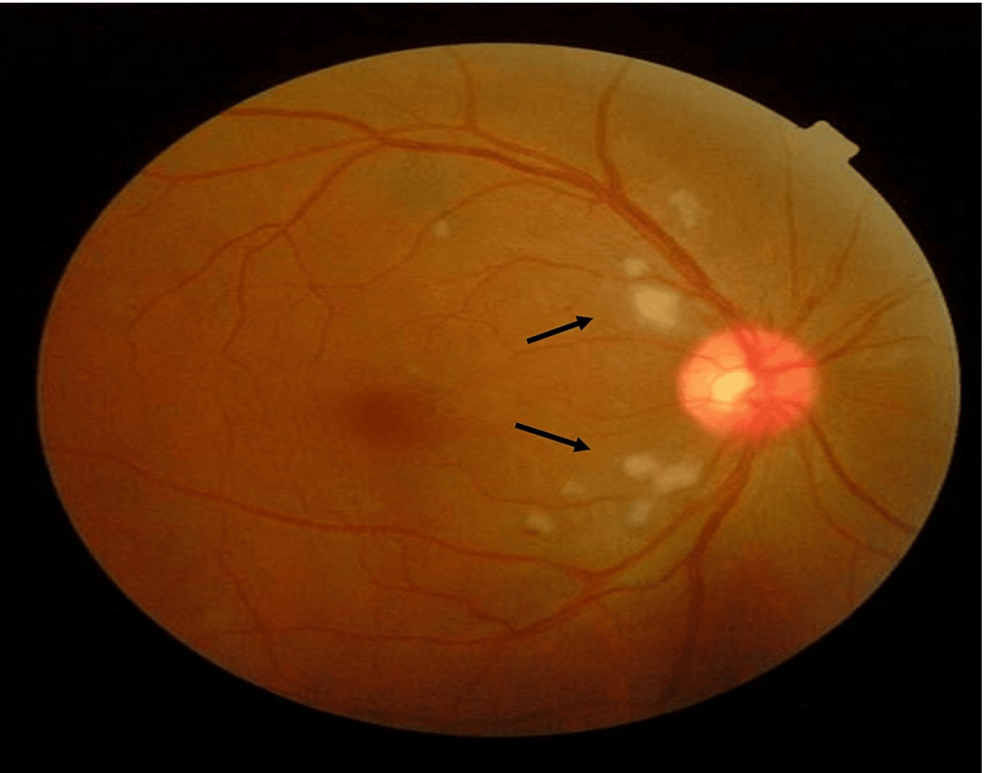
The patient's right eye fundus at initial presentation Right eye fundus showing few cotton wool spots and ‘flecken’ (black arrow) at the posterior pole

The visual acuity in the right eye was 6/12 (pinhole 6/12) while the left eye showed a visual acuity of 6/9. The pupillary assessment showed that the right eye had a negative relative afferent pupillary defect. Both anterior segments were unremarkable. Intraocular pressure in both eyes was normal. Fundoscopy showed multiple cotton wool spots and fleckens at the posterior pole on the right with a good fovea reflex (Figure 1).

The left fundus was normal. There were no signs of infective conditions, such as retinitis, vasculitis, vitritis, or choroiditis, and no retinal or vitreous hemorrhages, seen in the right eye.

**Figure 2:**
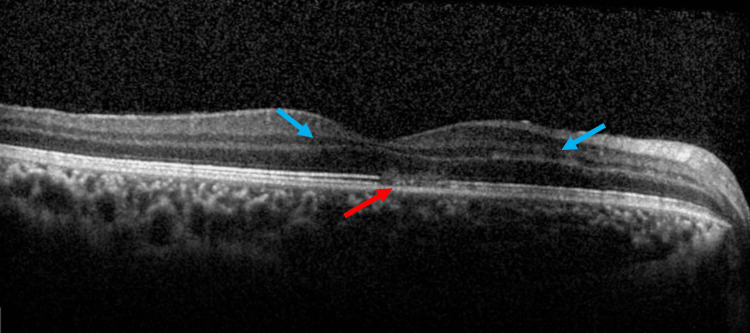
The patient's OCT of the right eye macula at initial presentation Optical coherence tomography (OCT) of the right eye macula displaying disruption of the photoreceptor (red arrow) and retinal edema (blue arrow) within the retinal nerve layer

Optical coherence tomography (OCT) of the right macula revealed photoreceptor disruption and retinal edema within the retinal layer (Figure 2).

The patient was diagnosed to have Purtscher’s retinopathy.

**Figure 3:**
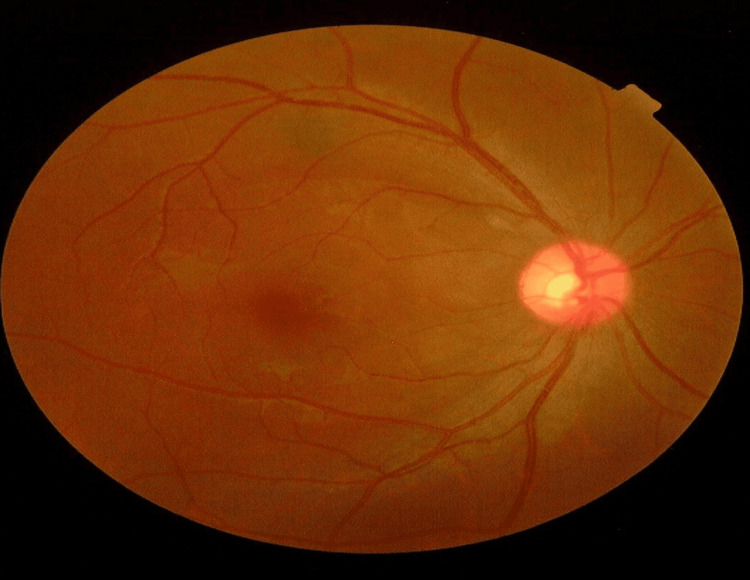
The patient's right eye fundus after one month Right eye fundus showing resolution of cotton wool spots and ‘flecken’ at the posterior pole

He was treated conservatively because his visual acuity was minimally affected. Upon discharge after one week, the visual acuity remained 6/12 in the right eye. At the one-month follow-up, the patient noticed there was an improvement in vision of his right eye. The visual acuity was 6/6 and the fundus showed complete resolution of the lesions (Figure 3).

**Figure 4:**
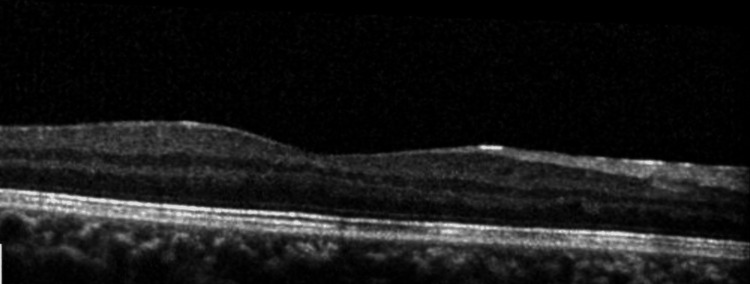
The patient's OCT of the right eye macula at one month Optical coherence tomography (OCT) of the right eye macula displaying resolution of photoreceptor disruption and retinal edema within the retinal nerve layer.

OCT revealed the resolution of disrupted photoreceptors and retinal edema within the retinal layer of the macula (Figure 4).

## Discussion

Purtscher’s retinopathy presents as an occlusive retinal microvasculopathy that poses a risk to vision. Typically associated with trauma, particularly head injury or thoracic compression, it manifests in affected patients’ post-injury [1,2]. Conversely, the designation 'Purtscher-like retinopathies' denotes individuals experiencing this condition in the absence of trauma but in conjunction with severe ailments like pancreatitis, renal failure, and autoimmune disorders [2]. Purtscher retinopathy is a traumatic retinal angiopathy or lymphorrhagia retinae or retinal teletraumatism. Purtscher documented abrupt vision loss in individuals with profound head injuries attributed to distant retinopathy. This condition is characterized by bilateral cotton wool spots, retinal hemorrhages, and optic disc swelling as observed through fundoscopy [8]. Similar retinopathy occurrences have been reported in cases of long bone fractures, acute pancreatitis, and compressive chest trauma [2,4]. Although Purtscher's flecken is regarded as pathognomonic, its occurrence is limited to only 50% of cases [9]. The predominant mechanism implicated in the pathogenesis of this condition is arteriolar occlusion resulting from embolization. Various substances, including air, fat, granulocytes, or aggregates of other blood products formed after complement activation, have all been proposed as potential emboli responsible for arteriolar occlusion [10,11]. In our case, the occlusion is most likely attributed to fat emboli, a consequence of a long bone fracture. Our report highlights a case of Purtscher’s retinopathy following a long bone fracture, which is rare compared to the other causes.

Purtscher's flecken is delineated as numerous discrete regions of retinal whitening located superficially in the inner retina, interspersed among arterioles and venules [2], these lesions predominantly concentrate within the posterior pole surrounding the optic disc, with rare extension beyond the mid-peripheral retina. Another unique presentation of this case was directed to unilateral involvement, as it is less well-recognized that Purtscher’s retinopathy can occur unilaterally [10]. Symptoms usually manifest within 48 hours, with patients presenting complaints of diminished visual acuity, frequently ranging from 6/60 to the ability to discern only finger counting. The findings in our patient are typical of Purtscher’s retinopathy.

According to Miguel et al., a diagnosis of Purtscher's retinopathy is established by the fulfillment of a minimum of three out of five predefined criteria [7]. These criteria include the presence of Purtscher's flecken, a low to moderate number of retinal hemorrhages (1-10), cotton wool spots, a likely or plausible explanatory etiology, and complementary investigations consistent with the diagnosis. Thus, our patient fulfills these criteria as he presented with cotton wool spots, and Purtscher’s flecken with a long bone fracture despite the absence of retinal hemorrhages.

Currently, there are no evidence-based guidelines regarding treating Purtscher’s retinopathy. Several authors have published case reports with different therapeutic approaches. The most reported treatment is high-dose systemic corticosteroids with successful results [12,13]. However, the efficacy of corticosteroids has not been established in prospective trials, and it remains controversial. In a study by Miguel et al., no statistically notable distinction in visual acuity enhancement was observed among patients administered corticosteroids compared to those without corticosteroid treatment [7]. Observation may be the most recommended therapeutic strategy, especially in patients with good visual acuity at presentation as in our case.

Typically, retinal lesions exhibit gradual resolution over several weeks, eventually restoring a normal retinal appearance, as evidenced in our observed case. However, in severe instances, the resolution may be accompanied by the development of pigmentary changes and optic atrophy. The foremost objective of treatment should prioritize the elimination of contributing factors. This involves the management of acute trauma or addressing underlying conditions such as pancreatitis, thereby mitigating the progression and complications associated with Purtscher's and Purtscher-like retinopathies.

## Conclusions

Purtscher’s retinopathy, an infrequent yet vision-endangering occlusive retinal microvasculopathy after trauma, mandates careful consideration. Trauma patients sustaining long bone fractures, head injuries, or chest compression necessitate meticulous assessment, particularly focusing on emerging visual symptoms. Urgent referral to an ophthalmologist is imperative upon symptom presentation for comprehensive ocular evaluation. This report details a case featuring unilateral Purtscher’s retinopathy, characterized by improved visual acuity and clinical resolution without necessitating therapeutic intervention. In cases of profound visual impairment, the effectiveness of corticosteroid therapy remains uncertain; however, it could be considered as a potential intervention.
